# Supplementary material for: Laboratory Performance Evaluation of a Low-Cost Electrochemical Formaldehyde Sensor
Source: Sensors (Basel). 2023 Aug 26;23(17):7444. doi: 10.3390/s23177444 (PMC10490822; doi:10.3390/s23177444)
Supplement: Supplementary file 1 [file sensors-23-07444-s001.zip › sensors-2576014-supplementary.pdf]

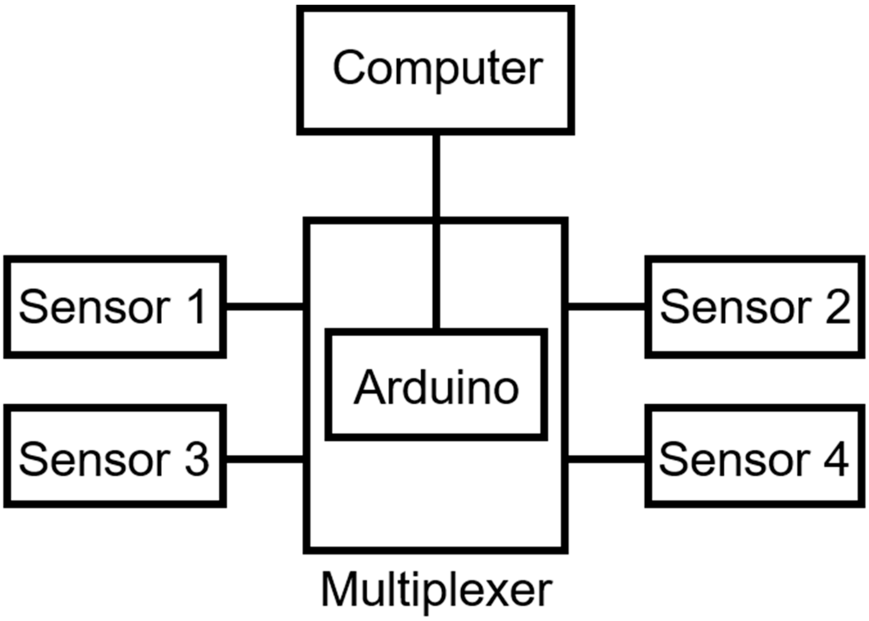

Figure S1. Sensor connections. Each sensor was placed in a closed 3D-printed PLA chamber.

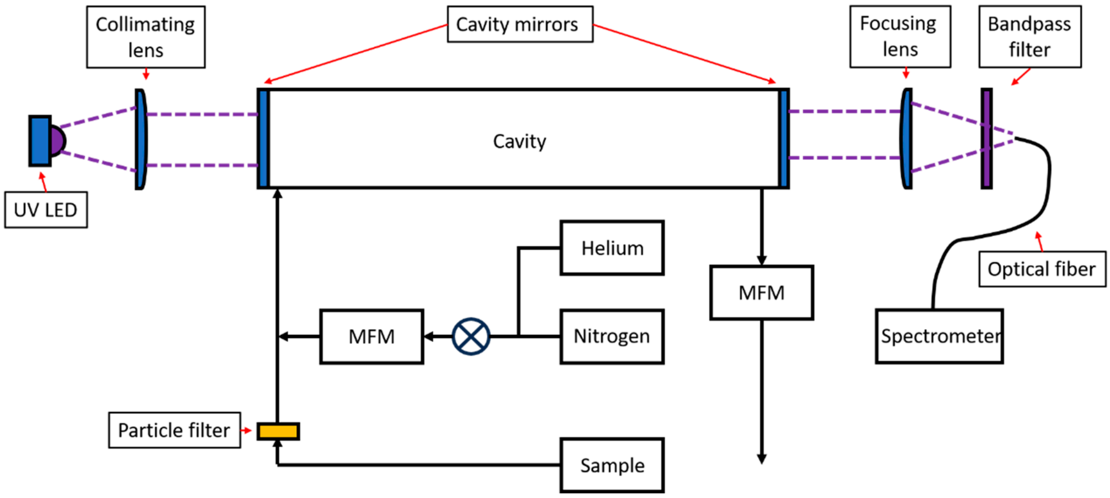

Figure S2. Schematic of the broadband cavity enhanced absorption spectrometer (BBCEAS) for formaldehyde measurement. Sample gas flows into the system and gas flow rate is measured by mass flow meters (MFM).

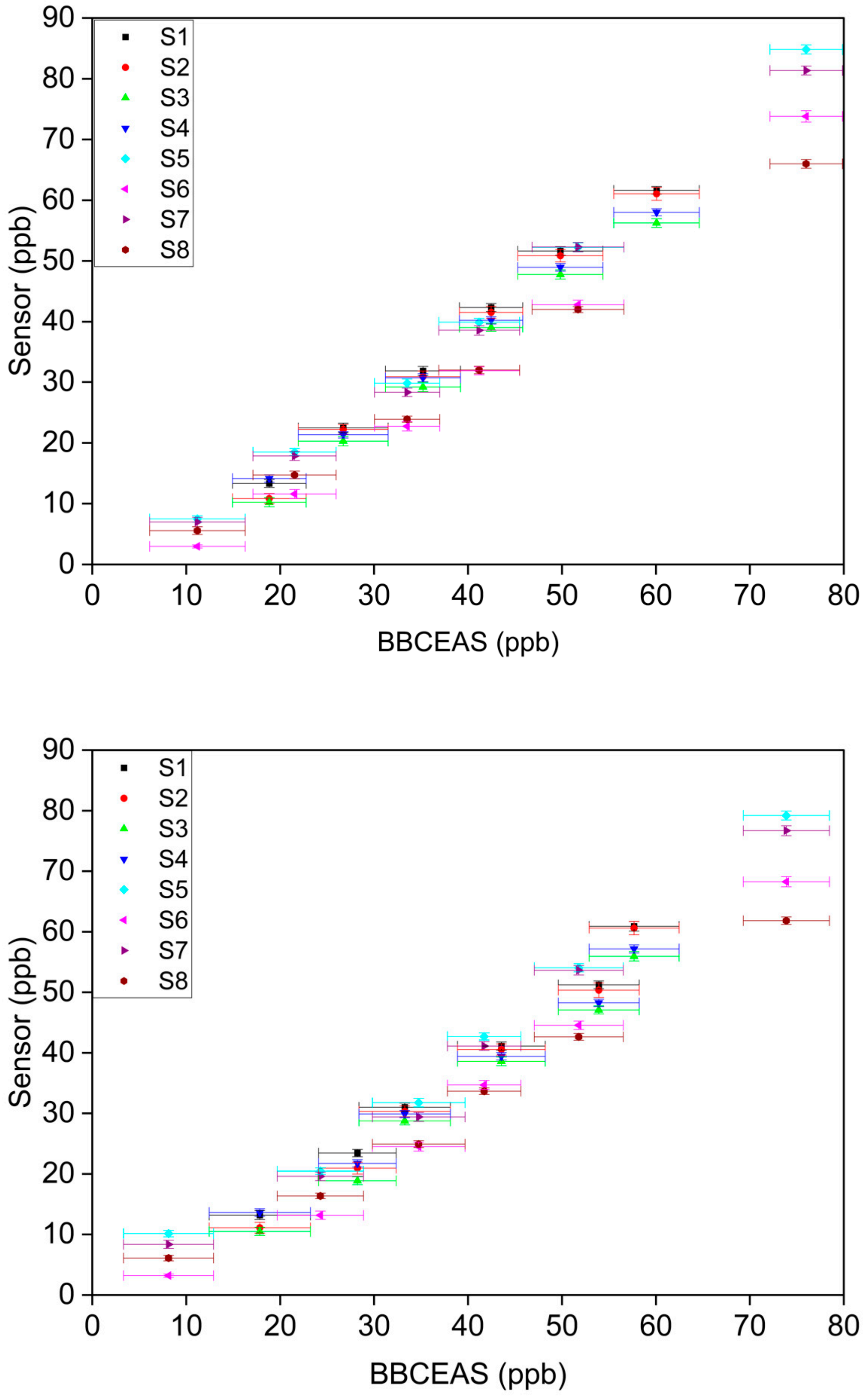

Figure S3. Response of eight sensors vs. the BBCEAS in the repeated concentration-only tests.

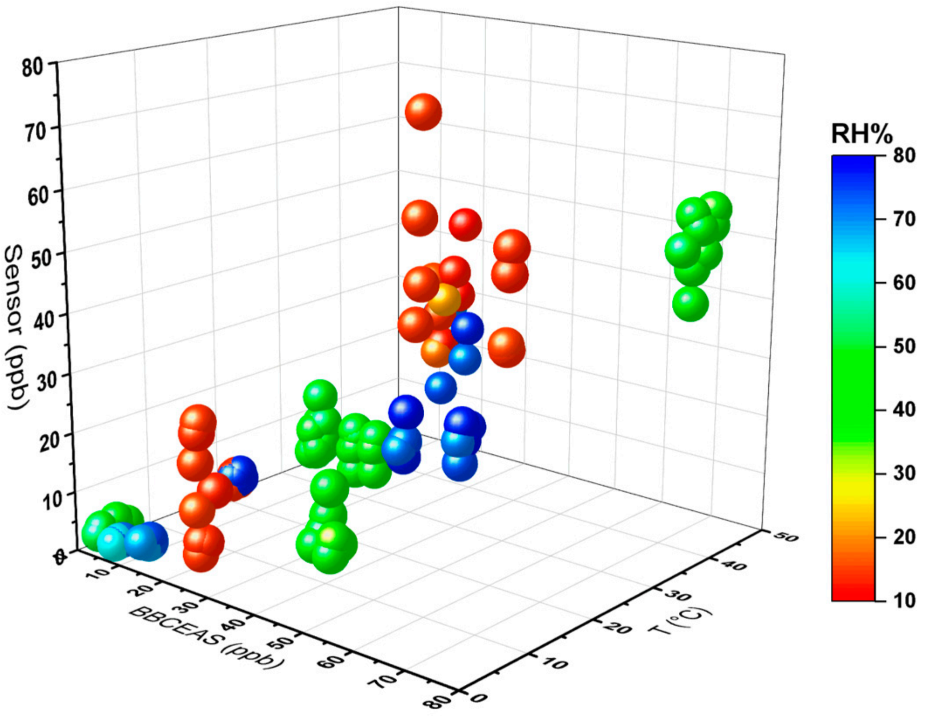

Figure S4. Sensor response vs. formaldehyde concentration, temperature, and RH. Experimental conditions selected by a Box-Behnken design. Note that this figure showed actual testing conditions.

Table S1. Actual conditions of the Box-Behnken experimental design

Sensor #	Formaldehyde concentration (ppb)	Temperature (℃)	Relative humidity (%)
S1	22.4±2.8	3.83±0.01	14.39±0.01
S1	26.6±3.5	38.24±0.03	22.19±2.20
S1	5.8±3.3	2.43±0.04	64.02±0.19
S1	21.6±6	41.02±0.04	75.68±0.01
S1	0.1±1.1	5.69±0.02	38.32±0.04
S1	2.6±3.1	39.86±0.26	42.86±0.33
S1	50.8±3.1	3.93±0.02	38.18±0.01
S1	75.0±7.1	40.97±0.02	46.32±0.03
S1	0.0±1.1	22.15±0.02	14.54±0.03
S1	0.1±1.3	22.31±0.03	75.71±0.25
S1	42.3±3.4	21.85±0.02	14.28±0.03
S1	39.2±3.8	21.66±0.01	72.30±0.05
S1	19.9±3.6	21.98±0.02	43.57±0.04
S1	21.1±3.3	21.80±0.03	45.26±0.14
S1	19.8±3.2	21.77±0.02	46.06±0.05
S2	22.4±2.8	3.46±0.02	15.00±0.01
S2	26.6±3.5	39.87±0.02	12.68±1.14
S2	5.8±3.3	2.92±0.04	71.04±0.15
S2	21.6±6	41.47±0.06	74.01±0.01
S2	0.1±1.1	4.81±0.02	45.45±0.06
S2	2.6±3.1	39.70±0.30	42.16±0.18
S2	50.8±3.1	3.42±0.02	46.13±0.02
S2	75.0±7.1	41.76±0.02	41.22±0.02
S2	0.0±1.1	22.76±0.02	14.45±0.03
S2	0.1±1.3	22.84±0.02	77.26±0.09
S2	42.3±3.4	22.45±0.02	14.50±0.02
S2	39.2±3.8	22.28±0.01	77.08±0.04
S2	19.9±3.6	22.57±0.01	46.95±0.02
S2	21.1±3.3	22.40±0.04	48.16±0.11
S2	19.8±3.2	22.37±0.02	48.57±0.07
S3	22.4±2.8	3.36±0.02	14.38±0.01
S3	26.6±3.5	41.69±0.01	10.95±0.02
S3	5.8±3.3	2.10±0.03	71.65±0.30
S3	21.6±6	43.42±0.05	71.10±0.03
S3	0.1±1.1	2.89±0.01	50.75±0.04
S3	2.6±3.1	40.87±0.34	42.80±0.15
S3	50.8±3.1	3.42±0.01	45.28±0.01
S3	75.0±7.1	43.19±0.01	41.02±0.12
S3	0.0±1.1	23.26±0.02	14.31±0.03
S3	0.1±1.3	23.41±0.03	76.78±0.09
S3	42.3±3.4	22.95±0.02	14.55±0.01
S3	39.2±3.8	22.73±0.02	78.17±0.04
S3	19.9±3.6	23.06±0.01	47.66±0.02
S3	21.1±3.3	22.91±0.04	48.72±0.10
S3	19.8±3.2	22.88±0.02	49.03±0.07
S4	22.4±2.8	3.93±0.01	13.93±0.01
S4	26.6±3.5	40.61±0.02	12.06±1.04
S4	5.8±3.3	1.84±0.03	61.73±0.36
S4	21.6±6	43.20±0.06	70.16±0.04
S4	0.1±1.1	4.31±0.04	40.42±0.05

S4	2.6±3.1	41.12±0.34	45.10±0.19
S4	50.8±3.1	4.28±0.02	36.45±0.01
S4	75.0±7.1	42.90±0.03	43.46±0.03
S4	0.0±1.1	23.07±0.03	14.01±0.04
S4	0.1±1.3	23.25±0.03	75.93±0.08
S4	42.3±3.4	22.70±0.02	14.23±0.03
S4	39.2±3.8	22.46±0.01	73.46±0.04
S4	19.9±3.6	22.86±0.02	44.50±0.03
S4	21.1±3.3	22.70±0.03	45.84±0.11
S4	19.8±3.2	22.66±0.02	46.45±0.06
S5	23.0±3.0	3.55±0.01	14.17±0.01
S5	23.2±3.4	40.04±0.08	14.70±0.66
S5	10.8±3.0	3.82±0.01	73.83±0.33
S5	28.2±5.8	40.97±0.02	77.10±0.06
S5	0.0±1.1	5.37±0.02	44.88±0.05
S5	2.6±3.1	40.09±0.13	44.37±0.41
S5	47.5±3.1	5.41±0.02	44.91±0.28
S5	73.7±4.6	40.96±0.04	45.26±0.51
S5	0.0±2.3	22.78±0.01	13.65±0.03
S5	0.8±7.0	23.05±0.02	77.15±0.03
S5	59.3±4.4	23.21±0.01	13.95±0.36
S5	50.6±3.4	22.90±0.02	77.64±0.05
S5	27.5±4.3	23.25±0.01	46.45±0.07
S5	28.0±4.3	23.25±0.01	47.20±0.02
S5	32.0±4.5	23.26±0.01	46.13±0.02
S6	23.0±3.0	4.26±0.01	13.53±0.02
S6	23.2±3.4	39.65±0.08	18.23±0.71
S6	10.8±3.0	3.62±0.03	68.08±0.43
S6	28.2±5.8	40.57±0.02	75.91±0.04
S6	0.0±1.1	6.77±0.01	39.07±0.04
S6	2.6±3.1	40.34±0.13	43.35±0.44
S6	47.5±3.1	6.84±0.02	37.35±0.46
S6	73.7±4.6	40.92±0.04	44.98±0.58
S6	0.0±2.3	22.50±0.02	13.43±0.05
S6	0.8±7.0	22.78±0.02	76.71±0.03
S6	59.3±4.4	22.92±0.01	14.22±0.09
S6	50.6±3.4	22.60±0.02	73.88±0.07
S6	27.5±4.3	22.96±0.01	43.87±0.30
S6	28.0±4.3	22.96±0.01	44.73±0.02
S6	32.0±4.5	22.98±0.01	43.84±0.02
S7	23.0±3.0	5.61±0.01	13.09±0.02
S7	23.2±3.4	39.00±0.08	16.05±0.76
S7	10.8±3.0	2.68±0.02	68.44±0.39
S7	28.2±5.8	40.55±0.02	72.61±0.06
S7	0.0±1.1	3.36±0.01	49.23±0.02
S7	2.6±3.1	39.46±0.12	45.45±0.43
S7	47.5±3.1	3.53±0.02	45.80±0.63
S7	73.7±4.6	39.21±0.06	48.35±0.37
S7	0.0±2.3	22.40±0.02	13.41±0.04
S7	0.8±7.0	22.64±0.02	76.64±0.02
S7	59.3±4.4	22.79±0.02	15.07±0.24
S7	50.6±3.4	22.45±0.01	71.28±0.07
S7	27.5±4.3	22.88±0.01	43.00±0.30

S7	28.0±4.3	22.88±0.02	43.86±0.01
S7	32.0±4.5	22.90±0.01	43.11±0.02
S8	23.0±3.0	3.29±0.02	13.61±0.01
S8	23.2±3.4	40.44±0.07	12.74±0.86
S8	10.8±3.0	3.88±0.02	72.93±0.34
S8	28.2±5.8	41.54±0.01	76.68±0.04
S8	0.0±1.1	4.63±0.01	47.07±0.03
S8	2.6±3.1	39.84±0.15	45.90±0.34
S8	47.5±3.1	4.72±0.02	46.87±0.33
S8	73.7±4.6	40.97±0.05	45.49±0.52
S8	0.0±2.3	23.04±0.01	13.40±0.04
S8	0.8±7.0	23.29±0.02	76.52±0.03
S8	59.3±4.4	23.46±0.02	13.61±0.41
S8	50.6±3.4	23.10±0.02	78.51±0.04
S8	27.5±4.3	23.54±0.01	46.56±0.07
S8	28.0±4.3	23.54±0.01	47.27±0.03
S8	32.0±4.5	23.55±0.01	46.16±0.02

Table S2. Evaluation metrics of sensors in the repeated concentration-only tests

Metrics	Test#	S1	S2	S3	S4	S5	S6	S7	S8
Slope	Test1	1.21	1.24	1.13	1.08	1.06	0.95	1.21	1.01
	Test2	1.20	1.23	1.14	1.10	1.19	1.10	1.16	0.93
	Test3	1.15	1.19	1.11	1.06	1.09	1.02	1.08	0.87
Intercept	Test1	-8.90	-10.86	-10.33	-6.49	-3.63	-8.26	-5.72	-4.90
	Test2	-9.34	-11.43	-10.36	-7.19	-7.92	-12.01	-7.74	-5.88
	Test3	-7.93	-10.87	-9.97	-6.26	-2.75	-8.54	-4.05	-3.09
R^2	Test1	0.986	0.990	0.991	0.984	0.994	0.994	0.995	0.995
	Test2	0.997	0.997	0.995	0.997	0.995	0.993	0.996	0.998
	Test3	0.989	0.986	0.986	0.987	0.987	0.989	0.987	0.993
LOD (ppb)	Test1	6.56	5.61	5.31	7.05	6.46	6.41	5.61	5.96
	Test2	2.86	3.24	3.92	3.29	5.89	7.16	5.40	3.92
	Test3	5.97	6.69	6.89	6.57	9.63	8.81	9.73	6.90
RMSE (ppb)	Test1	2.96	3.66	5.59	3.65	1.84	10.12	4.75	4.47
	Test2	3.19	4.08	5.40	3.57	4.05	8.47	3.66	8.60
	Test3	3.04	4.35	5.97	4.13	1.90	7.70	1.90	8.60
NRMSE	Test1	0.0763	0.0944	0.144	0.0942	0.0305	0.168	0.0789	0.0743
	Test2	0.0774	0.0991	0.131	0.0866	0.0625	0.131	0.0564	0.133
	Test3	0.0764	0.109	0.150	0.104	0.0289	0.117	0.0289	0.131
MAE (ppb)	Test1	2.52	3.08	5.30	3.50	1.59	10.07	3.79	4.47
	Test2	2.67	3.35	5.06	3.29	3.29	8.22	3.11	8.49
	Test3	2.40	3.46	5.78	4.06	1.53	7.69	1.57	8.16

Table S3. Coefficient of variation (*CV*) for the eight sensors in the repeated concentration-only tests

Sensor	S1	S2	S3	S4	S5	S6	S7	S8
CV (%)	1.36	1.74	1.60	1.21	6.41	7.37	3.94	4.04

Table S4. Sensor response time (t_{90}) in the concentration-only tests

BBCEAS concentration (ppb)	S1 (s)	S2 (s)	S3 (s)	S4 (s)
17.2	168	374	280	178
28.2	243	364	308	317
33.7	149	345	261	233
42.6	234	421	318	318
52.1	281	411	327	318
18.9	178	364	252	131
26.7	168	346	318	290
35.2	243	411	337	309
42.5	289	439	336	317
49.8	317	402	327	327
17.8	252	392	289	214
28.2	187	421	309	262
33.3	216	297	216	243
43.6	306	387	324	270
53.9	306	396	297	279
57.7	333	396	315	306

Table S5. R^2 and *RMSE* for the MLR model variable selection

R^2	C	C+T	C+T+RH	C+T+RH+intercept
group	0.813	0.845	0.878	0.751
S1	0.826	0.891	0.915	0.839
S2	0.777	0.810	0.863	0.754
S3	0.807	0.834	0.865	0.752
S4	0.819	0.881	0.908	0.813
S5	0.895	0.918	0.942	0.863
S6	0.868	0.880	0.927	0.854
S7	0.855	0.902	0.934	0.846
S8	0.868	0.870	0.915	0.864

RMSE (ppb)	C	C+T	C+T+RH	C+T+RH+intercept
group	11.85	12.57	13.28	13.32
S1	10.81	12.21	12.67	12.82
S2	10.30	11.16	12.42	12.61
S3	4.12	6.46	8.35	9.16
S4	9.94	11.53	12.15	12.19
S5	11.06	11.71	12.32	12.41
S6	18.80	18.89	19.26	19.26
S7	14.56	15.31	15.81	15.81
S8	14.04	14.08	14.85	15.21

Table S6. Variable coefficients of the MLR model

Variables ignoring intercept	Coefficients	*P*-value	95% CI
Intercept	0	N/A	N/A
C	0.552	1.01×10^{-31}	[0.485, 0.620]
T	0.399	3.06×10^{-12}	[0.298, 0.501]
RH	-0.142	1.02×10^{-7}	[-0.191, -0.092]
Variables including intercept	Coefficients	*P*-value	95% CI
Intercept	2.953	0.188	[-1.465, 7.371]
C	0.536	1.62×10^{-28}	[0.464, 0.607]
T	0.364	4.65×10^{-9}	[0.250, 0.478]
RH	-0.174	1.90×10^{-6}	[-0.242, -0.105]

Table S7. Preliminary cross-sensitivity tests

Interferent gas		CO	NO	NO_2	Isobutylene
Concentration		39.7±0.53 ppm	100.5±0.20 ppb	83.1±0.19 ppb	100±0.00 ppb
Sensor signal (ppb)	S5	1.10±0.11	1.41±0.05	0.20±0.00	1.99±0.14
	S6	0.58±0.06	0.99±0.05	0.20±0.00	1.23±0.08
	S7	1.23±0.08	1.45±0.09	0.20±0.00	1.82±0.10
	S8	1.56±0.12	1.30±0.10	0.20±0.00	1.61±0.04
P-value	S5	2.44×10^{-116}	2.73×10^{-17}	8.87×10^{-125}	0.876
	S6	7.04×10^{-143}	4.84×10^{-67}	6.43×10^{-176}	0.294
	S7	2.77×10^{-100}	1.83×10^{-19}	1.60×10^{-124}	0.193
	S8	4.03×10^{-5}	3.30×10^{-23}	7.24×10^{-124}	0.064

Note that the average sensor signal of zero air (baseline) is 1.66±0.30 ppb.
